# IFI27/ISG12 Downregulates Estrogen Receptor α Transactivation by Facilitating Its Interaction With CRM1/XPO1 in Breast Cancer Cells

**DOI:** 10.3389/fendo.2020.568375

**Published:** 2020-10-07

**Authors:** Mayte Guadalupe Cervantes-Badillo, Alejandro Paredes-Villa, Vania Gómez-Romero, Rafael Cervantes-Roldán, Luis E. Arias-Romero, Olga Villamar-Cruz, Miroslava González-Montiel, Tonatiuh Barrios-García, Alberto J. Cabrera-Quintero, Gabriel Rodríguez-Gómez, Laura Cancino-Villeda, Alejandro Zentella-Dehesa, Alfonso León-Del-Río

**Affiliations:** ^1^Programa de Investigación de Cáncer de Mama, Instituto de Investigaciones Biomédicas, Universidad Nacional Autónoma de México, Ciudad de México, Mexico; ^2^Unidad de Investigación en Biomedicina (UBIMED), Facultad de Estudios Superiores-Iztacala, Universidad Nacional Autónoma de México, Tlalnepantla, Mexico; ^3^Departamento de Medicina Genómica y Toxicología Ambiental, Instituto de Investigaciones Biomédicas, Universidad Nacional Autónoma de México, Ciudad de México, Mexico; ^4^Unidad de Bioquímica, Instituto Nacional de Ciencias Médicas y Nutrición Salvador Zubirán (INCMNSZ), Ciudad de México, Mexico

**Keywords:** ISG12, IFI27, estrogen receptor, CRM1, nuclear export, breast cancer

## Abstract

The estrogen receptor alpha (ERα) is a ligand-activated transcription factor whose activity is modulated by its interaction with multiple protein complexes. In this work, we have identified the protein interferon alpha inducible protein 27 (IFI27/ISG12) as a novel ERα-associated protein. IFI27/ISG12 transcription is regulated by interferon and estradiol and its overexpression is associated to reduced overall survival in ER+ breast cancer patients but its function in mammary gland tissue remains elusive. In this study we showed that overexpression of IFI27/ISG12 in breast cancer cells attenuates ERα transactivation activity and the expression of ERα-dependent genes. Our results demonstrated that IFI27/ISG12 overexpression in MCF-7 cells reduced their proliferation rate in 2-D and 3-D cell culture assays and impaired their ability to migrate in a wound-healing assay. We show that IFI27/ISG12 downregulation of ERα transactivation activity is mediated by its ability to facilitate the interaction between ERα and CRM1/XPO1 that mediates the nuclear export of large macromolecules to the cytoplasm. IFI27/ISG12 overexpression was shown to impair the estradiol-dependent proliferation and tamoxifen-induced apoptosis in breast cancer cells. Our results suggest that IFI27/ISG12 may be an important factor in regulating ERα activity in breast cancer cells by modifying its nuclear versus cytoplasmic protein levels. We propose that IFI27/ISG12 may be a potential target of future strategies to control the growth and proliferation of ERα−positive breast cancer tumors.

## Introduction

The estrogen receptor α (ERα) is a ligand-activated transcription factor that mediates the effects of the hormone estrogen (17β-estradiol; E2) on cell proliferation and differentiation in mammary gland and participates in maintenance of skeletal system, metabolic homeostasis and in development of central nervous system (1). In humans, ERα activity is also associated to the development, progression and metastasis of 70–80% of all breast cancer tumors (2). ERα has also been shown to play a key role in mediating resistance to apoptosis, immunosurveillance and hormone treatment in breast cancer (3–5). ERα belongs to the family of transcription factors known as nuclear hormone receptors and its structure is characterized for possessing functionally independent domains that include a DNA-binding domain, ligand-binding domain and two transactivation domains designated AF1 and AF2 that are located at the N-terminal and C-terminal regions, respectively (6–8). The functional synergistic interaction between the unique transcriptional properties of AF1 and AF2 domains is responsible for the full ligand-dependent ERα transactivation.

Mechanistically, the binding of E2 to ERα produces a major structural rearrangement on its ligand binding domain that allows AF2 to interact with a large array of coactivator proteins that include SRC-1, SRC-2/GRIP1/TIF2/NCoA2, SRC3/RAC3/p/CIP/ACTR/AIB1, CREB-binding protein (CBP)/p300, and CBP-associated factor (P/CAF) that increase ERα transcriptional activity by relaxing the chromatin structure through their histone acetyl-transferase activity (9–11). In the absence of E2 or in the presence of ERa antagonists, such as tamoxifen (TOT), the AF2 domain recruits corepressor proteins including NCoR, SMRT, and histone deacetylases (HDACs) that increase the condensation status of chromatin (10–12). Functional and molecular studies have also identified a large number of AF1 specific coregulators including BTF3 (basal transcription factor 3), SRA1 (steroid receptor RNA activator), CoCoA (coiled-coil coactivator), hMMS19 (human homolog of the yeast nucleotide excision repair gene MMS19), SPBP (stromelysin-1 platelet-derived growth factor-responsive element-binding protein), Smad4, NHERF2, and Tristetraprolin (TTP) (13–23). The exchange of coactivators and corepressors is a mechanism that finetunes the ERα transactivation activity in hormone responsive tissues and allows this transcription factor to oscillate between its functions as activator and repressor of gene expression (24, 25).

The transactivation activity of ERα can also be modulated through its interaction with proteins that affect its cellular localization. In its unliganded form ERα binds to an Hsp90 chaperone protein complex, which keeps ERα in a ligand-binding competent but inactive state that prevents it from binding to estrogen-response elements in the DNA (26–28). Some cell factors affect ERα transcriptional activity by regulating its mRNA and protein expression levels or its translocation in and out of the cell nucleus (3, 29–33).

In recent years the study and characterization of nuclear receptor associated proteins have been an important target for molecular oncology because dysregulation of their cellular expression levels has been associated with different forms of cancer. For example, changes in protein levels of coregulators SRC-1; NHERF2, TTP have been shown to correlate with tumor proliferation, disease recurrence or poor disease-free survival in breast cancer (19, 20, 34). Similarly, changes in the expression levels of proteins that affect the nucleocytoplasmic translocation of ERα such as CRM1/XPO1 or prosaposin have also been linked to breast cancer development (3, 31, 35).

In this work, we screened a yeast two-hybrid library to identify new ERα-associated proteins. Our studies identified a 122-amino acid protein, previously identified as interferon/estradiol induced p27/IFI27/ISG12 (hereafter ISG12) and which is overexpressed in breast cancer cells. Expression of ISG12 in MCF-7 cells down-regulates ERα transactivation activity and transcription of its target genes suggesting it functions as a nuclear receptor corepressor. However, immunohistochemistry and Western blot analysis of MCF-7 cells showed that ISG12 expression, unlike bona fide corepressors, such as NCoR and TTP, does not co-localize with nuclear DNA but it is confined to the nuclear envelope and cytoplasm. We demonstrate that the effect of ISG12 on ERα transactivation is mediated by enhancing the interaction between ERα and nuclear exportin CRM1/XPO1. We show further that ISG12 overexpression reduces proliferation and migration of MCF-7 cells and their ability to form spheroids in 3-D culture assays. We propose that ISG12 plays a role in the control of ERα transactivation by participating in the regulation of its protein levels in the cell nucleus of breast cancer cells.

## Materials and Methods

### Reagents and Antibodies

Estradiol (17 β-estradiol) and geneticin (G418) were from Sigma-Aldrich. Lipofectamine 2000 was purchased from Invitrogen. Human ERα and CRM1 antibodies were purchased from Santa Cruz Biotechnology, anti-FLAG antibody was from Sigma-Aldrich, and IFI27/ISG12 polyclonal antibody was purchased from Abcam.

### Plasmids

pcDNA3.1- ERα and 2XERE-Tk-LUC vectors have been previously described (19, 20). Human full-length IFI27/ISG12 mRNA (GenBank TM accession no. NM_001130080) was amplified by RT-PCR and cloned into the mammalian expression vector pCMV-3Tag-1A (Agilent Technologies, Santa Clara, CA). The resulting vector is referred as pCMV-3Tag-ISG12.

### Yeast Two-Hybrid Assay

The yeast two-hybrid screen was performed using matchmaker two-hybrid system kit (CLONTECH). Briefly, a cDNA fragment encoding the AF-1 domain (amino acids 1–180) of ERα was cloned into pAS2.1 vector to be used as a bait. A human mammary gland cDNA library in pACT2 plasmid was cotransformed with construct pAS2.1/AF1 into Y190 yeast cells. Yeast cells were plated on medium lacking tryptophan, leucine and histidine (SD/-Leu -Trp -His) containing 25 mM 3-amino-1,2,4-triazole(3-AT) and incubated for 2 to 4 days at 30°C. Resulting colonies were assayed for β-galactosidase activity. The positive AD plasmids were transformed into Escherichia coliDH5α cells for DNA sequencing and identification using Basic Local Alignment Search Tool (BLAST) analysis.

### Cell Culture and Transfection Assays

The luminal A breast cancer cell lines MCF7, T47D, and ZR-75-1 were obtained from the American Type Culture Collection (Manassas, VA, USA) and maintained in Dulbecco’s modified Eagle’s medium supplemented with 5% (v/v) inactivated fetal bovine serum (FBS) (GIBCO, Rockville MD, USA), 100 units/ml penicillin, and 100 μg/ml streptomycin (GIBCO, Rockville MD, USA) in a humidified atmosphere containing 5% CO2 at 37°C. Cells were grown in tissue culture dishes containing phenol red-free DMEM supplemented with 5% charcoal/dextran-treated FBS and cultured for 24 h before all experimental treatments with 100 nM E2. For transient transfection assays cells were grown to 80% confluence in 96 well plates and then they were transfected using Lipofectamine 2000 (Invitrogen), with 50 ng of ERE-TK-Luc, and 100 to 300 ng pCMV-3Tag-ISG12 vector or empty vector. Luciferase activity was determined using Dual-Glo Luciferase Assay System Protocol (Promega) according to the manufacturer’s instructions. For stable cell line transfection the ISG12 cDNA was subcloned into the pCMV-3Tag vector and the resulting pCMV-3Tag-ISG12 construct was transfected into MCF-7 cells using Lipofectamine 2000. The MCF7-ISG12 cells were plated in p150 plates containing G418 (500 μg*/*mL). G418-resistant cells were transferred to 96 well plates to select individual clones in the presence of G418. The MCF7-ISG12 clone used in this study was selected after confirming ISG12 over-expression by Western blot analysis. The effect of TOT and ISG12 on cell viability was determined using PrestoBlue (Thermo Fisher Scientific) following the manufacturer recommendations.

### Proximity Ligation Assays

To analyze the interaction between endogenous ERα and ISG12 proteins *in situ*, we used the Duolink Proximity Ligation Assay (PLA) (Sigma-Aldrich) in MCF-7, T47-D, and ZR-75-1 cells following the manufacturer’s instructions. Briefly, cells were grown on eight-well chamber slides (Lab-Tek) and stimulated with E2 for 1 h. The cells were fixed in 4% PFA in PBS, permeabilized in PBS-Triton X-100 0.05%, incubated in blocking solution for 1 h at 37°C and then in a solution containing mouse monoclonal anti-ERα antibody (D12, Santa Cruz Biotechnology) and rabbit anti-ISG12 antibody (Abcam) for 1 h at 37°C. The PLA probes consisting of secondary antibodies conjugated with complementary oligonucleotides were incubated for 1 h at 37°C. The ligation of the oligonucleotides was performed for 100 min at 37°C followed by an amplification step. Samples were analyzed under fluorescence microscopy using a Zeiss LSM710 Duo confocal microscope. Image acquisition was performed by imaging DAPI staining at a fixed Z Position while a Z stack of ± 5 μm at 1 μm intervals was carried out. The final image was stacked to a single level before further quantification. On each sample, at least three different fields were analyzed and fifty cells were counted in each. Results were represented as mean ± S.E. Significance (p-value) between cell lines was determined using the Student t-test. * p < 0.05; ** p < 0.01.

### 3-D Cell Cultures

For 3-D cultures 5000 control MCF-7 cells or MCF-7 cells overexpressing ISG12 were plated atop reconstituted basement membrane (Matrigel, Corning) in eight-well chamber slides as previously described (36). Cells were treated with vehicle (control), 100 nM 17 β-estradiol, or 1 µM Tamoxifen on day 14 and fixed on day 15. The 3-D cell cultures were then stained with Oregon Green Phalloidin (Thermo Fisher Scientific); 4',6 diamidino-2-phenylindole (DAPI) (Thermo Fisher Scientific) and anti–Ki-67, or anti-cleaved PARP1 (asp214) antibodies (Cell Signaling Technology). Samples were analyzed under fluorescence microscopy using a Zeiss LSM710 Duo confocal microscope. Percentage of Ki-67–positive, and anti-cleaved PARP1–positive cells were scored on the basis of assessment of 30 spheroids per well. Bar, 50 mm. Results were represented as mean ± S.E. Significance (p-value) between cell lines was determined using the Student t-test. * p < 0.05; ** p < 0.01.

### Wound Healing Assay

MCF-7 and MCF7-ISG12 cells were cultured to confluence in 6-well plates. The cell cultures were wounded with a sterile 10ul pipette tip, and then washed with PBS to remove floating cells. The cells were incubated with serum-free medium supplemented with 100 nM E2 for 24 or 48 h. Micrographs were taken and used to measure the migration of the cells. Cell migration into the wound surface was considered as the process of *in vitro* healing. Cell migration was calculated with the formula: (A_0_ − A_t_)/A_0_ × 100%, where A_0_ represents the area of the wound at 0 h, and A_t_ represents the area of the wound at 24 or 48 h.

### Immunoprecipitation and Western Blot

MCF-7 and MCF7-ISG12 cells were lysed with TNTE buffer (50 mM Tris-HCl, pH 7.4, 150 mM NaCl, 5 mM EDTA containing 0.5% Triton X-100 plus a mixture of protease inhibitors). Proteins were immunoprecipitated with rabbit polyclonal anti-ERα (HC-20) or mouse monoclonal anti-CRM1 (C-1). Immunoprecipitated proteins were separated by PAGE and detected by WB with mouse monoclonal anti-ER (D-12) or anti-CRM1 antibodies. Proteins were visualized by incubation with anti-rabbit or anti-mouse secondary horseradish-peroxidase-conjugated antibodies (Pierce, Thermo Fisher Scientific Inc.) and using an enhanced chemiluminescence assay (SuperSignal West Pico Chemiluminescent Substrate, Thermo Scientific).

### Immunofluorescence and Confocal Microscopy Studies

The cellular localization of ERα and ISG12 was determined by indirect immunofluorescence microscopy. Briefly, MCF-7 cells were grown on glass coverslips and fixed with freshly prepared 2% paraformaldehyde solution. The cells were incubated first with primary antibodies and then with secondary antibodies conjugated with Alexa-546 (red) and Alexa-488 (green; both from Molecular Probes, Eugene, OR). Prolong-Gold Antifade reagent with DAPI (blue; Invitrogen) was used to counterstain the DNA. Confocal analyses were performed using the Leica TCS SP8 confocal microscopy system and MRC600 laser-scanning confocal microscope (Bio-Rad, Hercules, CA). Each slide was examined at three excitation wavelengths (488, 546 and 633 nm). Quantification of nuclear ERα immunofluorescent signal (ERα signal/area) in control MCF-7 and MCF7-ISG12 cells is represented as mean ± SE. of three independent experiments (25–120 nuclei, each). Statistical significance (p value) for differences between MCF-7 and MCF7-ISG12 cells is shown as p < 0.05.

### RNA Isolation and RT‐PCR Analysis

Total RNA was isolated using Trizol Reagent (Invitrogen, Carlsbad, CA, USA) according to the manufacturer’s protocol. RNA quality was assessed using spectrophotometric methods and formaldehyde‐agarose gel electrophoresis, considering the 28S/18S rRNA ratio. Two micrograms of total RNA were DNase I (RNase‐free) treated (Ambion, Austin, TX, USA). cDNA synthesis was performed using SuperScript II Reverse Transcriptase (Invitrogen), following the manufacturer’s protocol. Quantitative PCR amplification was carried out using Maxima SYBR Green/ROX qPCR Master Mix (2×) (Thermo Fisher Scientific) and the following primers: GREB1 Fw 5'-CAAAGAATAACCTGTTGGCCCTGC-3', GREB1 Rv 5'-GACATGCCTGCGCTCTCATACTTA-3'; CTSD Fw 5'-CCCTCCATCCACTGCAAACT-3', CTSD Rv 5'TGCCTCTCCACTTTGACACC-3', GAPDH Fw 5'-AGCCACATCGCTCAGACAC-3', GAPDH Rv 5'-GCCCAATACGACCAAATCC-3'. Data were measured with the LightCycler^®^96 system (Roche Diagnostics International Ltd.). Expression of individual genes was compared and normalized using the 2-ΔΔCt method against the level of GAPDH mRNA.

### Cell Proliferation Analysis

Dynamic monitoring of cell proliferation was performed with the xCELLigence™ System (Acea Biosciences, San Diego CA, USA). MCF7 and MCF7-ISG12 cells were grown at a density of 7.5 × 10^3^ cells/well in quadruplicate on an E-plate 16 using phenol red-free DMEM supplemented with 5% charcoal/dextran-treated FBS. When the cell cultures reached a cell index of 0.5 the medium was supplemented with vehicle (ethanol 0.01%) or 10 nM E2. Cell growth curves were recorded on the xCELLigence™ RTCA System in real-time every 30 min, for at least 96 h.

### ISG12 mRNA Expression Levels in Breast Cancer Tumors and Normal Tissue and Kaplan-Meier Analysis

To compared ISG12 mRNA levels in breast cancer tumors and normal tissue we made use of the Breast Cancer Gene-Expression Miner database (http://bcgenex.centregauducheau.fr/BC-GEM/GEM-Accueil.php?js=1). The results are shown as a violin plot of the log2 of ISG12 mRNA expression (p=0.0001, Dunnett-Tukey-Kramer’s test). Relapse free survival (RFS) plots were generated using the gene chip database Kaplan-Meier Plotter (https://kmplot.com).The survival analysis was restricted to ERa status and tamoxifen vs other endocrine treatments. Logrank P < 0.05 was considered as statistically significant.

### Statistics

The experiments were performed in triplicate and presented as mean ± SD. Student’s t-test with the GraphPad prism 8 software were used for statistical analyses. P<0.05 was considered as statistically significant.

## Results

### Identification of ISG12 as an ERα Interacting Protein

To identify cell proteins that recognize ERα, we used its AF1 domain (amino acids 1–180) as bait in a yeast two-hybrid screen of 5 × 10^6^ independent clones of a human mammary gland cDNA library. The cDNA clones isolated from the cDNA library screen were subcloned into the pCMV-3Tag vector and co-transfected into MCF-7 cells with the reporter vector ERE-TK-Luc. Two independent clones had an open reading frame encoding a 122 amino acid protein. Sequence analysis using the BLAST program of the National Center for Biotechnology Information showed that the candidate protein had been previously described by different groups as interferon-inducible protein 27 P27/IFI27/ISG12, hereafter ISG12 (37–39).

**Figure 1:**
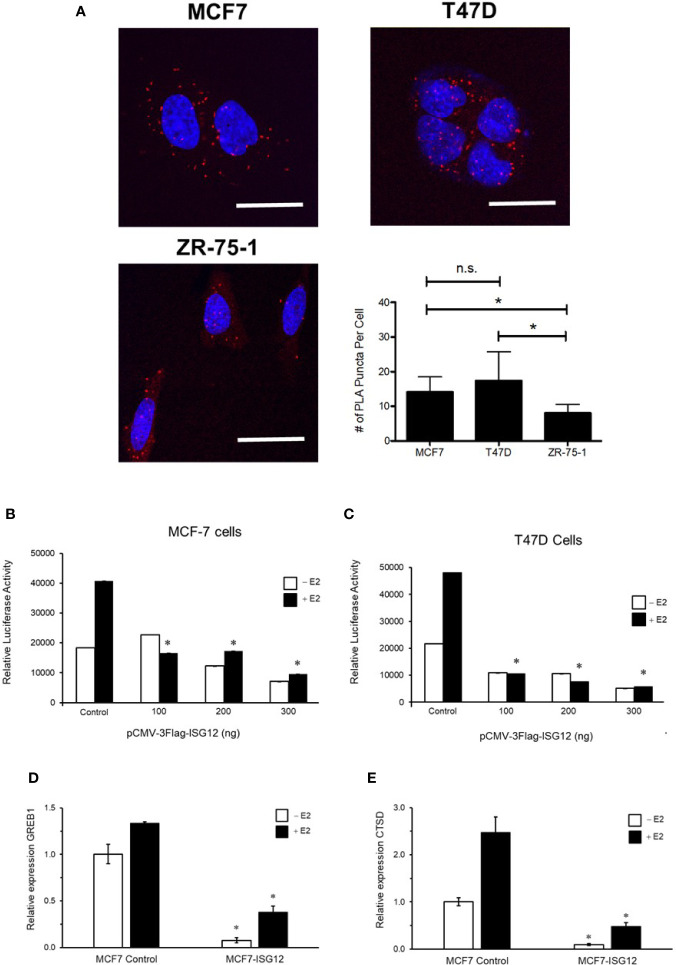
Proximity ligation assays and effect of transient expression of ISG12 on ERα transactivation activity. **(A)** The physical interaction between endogenously expressed ERα and ISG12 in MCF-7, T47-D and ZR-75-1 cells was determined using proximity ligation assays. The figure shows representative confocal microscopy images in which ERα-ISG12 interactions appear as an individual fluorescent red dots. Scale bar, 50 μm. Quantification of ERa-ISG12 interactions in MCF-7, T47-D, and ZR-75-1 cells is represented as mean ± S.E. of two independent experiments. The average number of ERα-ISG12 interactions in ZR-75-1 cells was found to be statistically significant (p < 0.05) when compared to the results obtained in MCF-7 and T47-D cells. MCF-7 **(B)** and T47D **(C)** cells were transiently transfected with empty pcDNAvector (control) or with different concentrations (100, 200, 300 ng) of pCMV-3TAG-ISG12 along with ERE-Tk-LUC reporter vector. The effect on ERα transactivation was determined by assay of luciferase activity. Assays were performed in triplicate in three independent experiments in the presence (white bars) or absence (black bars) of E2, and the results are represented as mean ± S.E. of three independent experiments. ISG12 reduces GREB1 **(D)** and CTSD **(E)** mRNA levels. MCF-7 cells were transfected with or without pCMV-3TAG-ISG12 and were stimulated with or without E2 for 24 h. Total RNA isolated from these cell cultures was used to determine GREB1 and CTSD mRNA levels by qPCR. GADPH mRNA was used as an expression control. Results are represented as mean ± S.E. (error bars) of three independent experiments. Differences in ERα activity and GREB1 and CTSD mRNA levels between control MCF-7 cells and MCF7-ISG12 cells were shown to be statistically significant (p < 0.05) *p < 0.05; n.s., not statistically significant.

To confirm ISG12 is an ERα-associated protein in human cells *in vivo*, we used the technique proximity ligation assay (PLA). Physical interaction between endogenous ERα and ISG12 proteins was determined in human breast cancer cell lines MCF-7, T47D, and ZR-75-1 using the corresponding two primary antibodies raised in different species. Next, the cells were incubated with species-specific secondary antibodies attached to a unique DNA strand (PLA probes). If the PLA probes are located less than 40 nm apart in the cell, the DNA strands can interact forming a circle that can be amplified by DNA polymerase. Hybridization with complementary fluorescent oligonucleotide probes allows the visualization of ERα-ISG12 interactions as an individual fluorescent red dot. The results revealed multiple loci of interactions between endogenous ERα and-ISG12 in the cytoplasm, nucleus and perinuclear region in MCF-7, T47D, and ZR-75-1 cells (**Figure 1A**). Quantification of the number of ERα-ISG12 interaction dots showed no significant difference between MCF-7 and T47-D cells. However, the number of ERα-ISG12 interaction events in ZR-75-1 cells was 50% and 60% lower (p < 0.05) than in MCF-7 and T47-D cells, respectively (**Figure 1A**).

### ISG12 Down-Regulates ERα Transcriptional Activity

To explore the effect of ISG12 expression on ERα transactivation, we performed transient transfection assays in MCF-7 and T47-D cells. In these experiments, pCMV-3Tag-ISG12 was the source of ISG12, and the vector 2xERE-Tk-LUC was used as the indicator of ERα transcriptional activity. In the two cell lines the baseline luciferase activity increased upon E2 stimulation (**Figures 1B, C**, control). ISG12 overexpression in MCF-7 and T47D cell lines produced a dose-dependent down-regulation of ERα transactivation activity. Transfection with 100, 200 or 300 ng of pCMV-3Tag-ISG12 in the presence of E2 reduced the transcriptional activity of ERα by 60%, 58% and 77% in MCF-7 cells (p < 0.05) (**Figure 1B**). The same treatment reduced E2-stimulated ERα activity by 79%, 84%, and 88% in T47D cells (p < 0.05) (**Figure 1C**). In the absence of E2, transfection of increasing amounts of ISG12 also reduced the basal ERα activity by 65% and 77% in MCF-7 and T47D cells, respectively (**Figures 1B, C**). To confirm the functional impact of ISG12 in the transcriptional activity of ERα we used qPCR to determine the mRNA levels of GREB1 and cathepsin D (CTSD) in control MCF-7 and ISG12-overexpressing MCF-7 cells incubated in hormone-free medium or in medium supplemented with E2. Control MCF-7 cells stimulated with 100 nM E2 exhibited a 33% increase in GREB1 mRNA levels and 147% increase in CTSD mRNA levels compared to unstimulated MCF-7 cells (**Figure 1D**, MCF-7 control). In contrast, ISG12-overexpressing MCF-7 cells exhibited 90% and 67% reduction in GREB1 mRNA levels in hormone-free and E2-medium with respect to control MCF-7 cells (**Figure 1D**, MCF7-ISG12). CTSD mRNA levels in MCF7-ISG12 cells were also reduced by 90% and 80% in hormone-free and E2 medium, respectively (p < 0.05) (**Figure 1E**, MCF7-ISG12). In combination, the PLA and transient transfection assays suggest that in human breast cancer cells ISG12 is an ERα associated protein and that its expression down-regulates the transactivation of this hormone nuclear receptor. Based on the similarities of the PLA and transient transfection results obtained in the different breast cancer cell lines tested we decided to continue the characterization of ISG12 as an ERα-associated protein using a MCF-7 cell line stably transfected with ISG12 (MCF7-ISG12).

### ISG12 Reduces ERα Protein Levels in MCF-7 Cells

**Figure 2:**
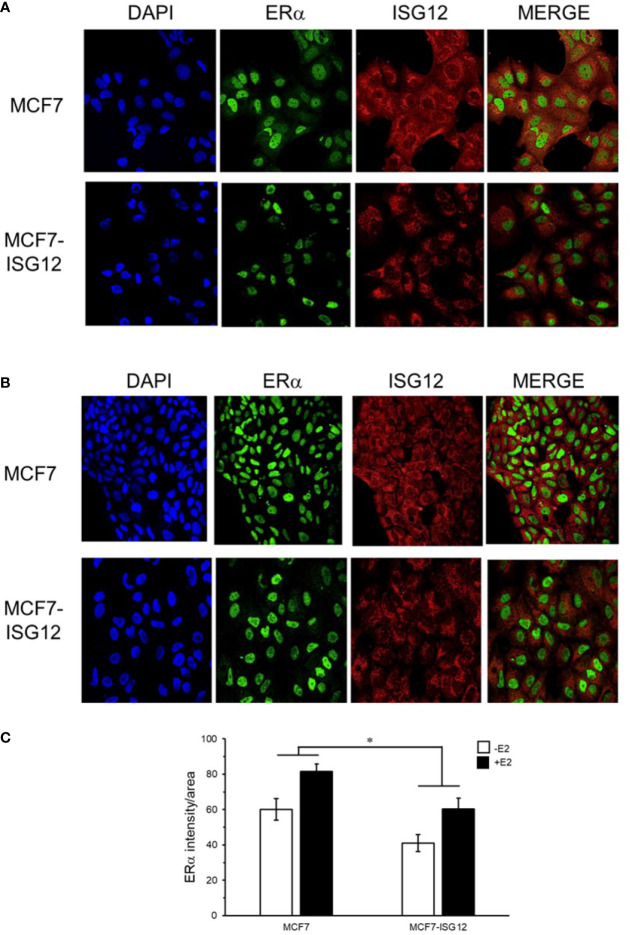
Subcellular localization of ISG12 and ERα in MCF-7 cells and MCF7-ISG12. Control MCF-7 and MCF7-ISG12 cells were cultured in the absence **(A)** or presence **(B)** of E2. The cultures were treated with DAPI to visualize nuclear DNA (blue, panel), anti-ERα antibody (green), and anti-ISG12 antibody (red) as described under *Material and Methods*. **(C)** Quantification of nuclear ERα immunofluorescent signal (ERα signal/area) in control MCF-7 and MCF7-ISG12 cells is represented as mean ± S.E. of three independent experiments. Differences between MCF-7 and MCF7-ISG12 cells were shown to be statistically significant (*p < 0.05).

**Figure 3:**
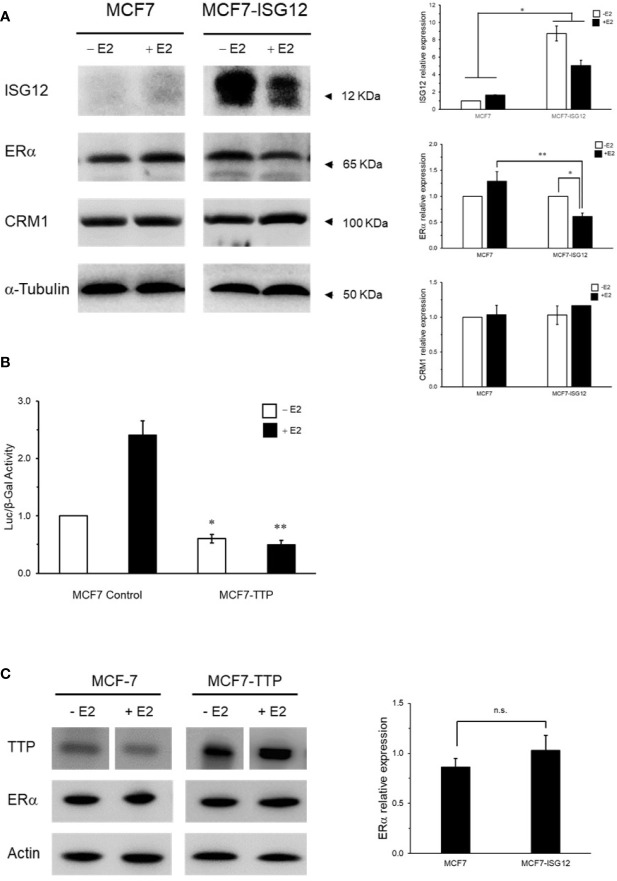
ISG12 and ERα protein levels in breast cancer cells. **(A)** Total protein extracts from control MCF-7 and MCF7-ISG12 cells grown in absence (−E2) or presence (+E2) were resolved by PAGE, and expression levels of ISG12, ERα, nuclear exportin CRM1/XPO1, and α-tubulin, as a loading control protein, were evaluated by Western blot using specific antibodies. Densitometric analysis of Western blot bands is represented as mean ± S.E. of three independent experiments (*p < 0.05, **p < 0.01). **(B)** The nuclear receptor corepressor TTP was used as a control to determine the specificity of the ISG12 effect on ERα. MCF-7 or MCF-7 over-expressing TTP (MCF7-TTP) were transfected with ERE-Tk-LUC reporter vector incubated in absence (white bars) or presence (black bars) of E2 and ERα transcriptional activity was determined as described in *Material and Methods*. Results are represented as mean ± S.E. of two different experiments and differences in ERα activity between control MCF-7 cells and MCF7-ISG12 cells were shown to be statistically significant (p < 0.05). **(C)** Total protein extracts from control MCF-7 and MCF7-TTP cells grown in absence (−E2) or presence (+E2) were resolved by PAGE, and expression levels of TTP, ERα and actin, as a loading control protein, were evaluated by Western blot using specific antibodies as described in *Material and Methods*. Western blot ERα protein bands from control MCF-7 and MCF7-ISG12 cells grown in the presence of E2 were analyzed by densitometry and the results are represented as mean S.E. of three independent experiments.

To continue the characterization of the effect of ISG12 on ERα transactivation, we determined its cellular localization with respect to ERα in control MCF-7 cells and MCF-7 cells stably transfected with pCMV-3Tag-ISG12 incubated in hormone-free medium (**Figure 2A**) or in medium containing E2 (**Figure 2B**). Immunostaining of MCF-7 and MCF7-ISG12 cells with anti-ERα antibody (green) showed that ERα is predominantly localized in the cell nucleus and it seems to be more abundant in the nucleus of cells incubated in the presence of E2 (**Figures 2A, B**, ERα panel). Incubation of MCF-7 and MCF7-ISG12 cells with anti-ISG12 antibody (red) demonstrated ISG12 is expressed in the cytoplasm and nuclear envelope but it seems to be less abundant inside the cell nucleus (**Figures 2A, B**, ISG12 panel). However, MCF7-ISG12 cells exhibited lower nuclear ERα protein levels compared to control MCF-7 cells. Quantification of the ERα signal/nuclear area showed that in MCF7-ISG12 cells the ERα immunofluorescent signal is reduced by 34% and 26% with respect to control MCF-7 cells (p < 0.05) incubated in hormone-free and E2 supplemented medium, respectively (**Figure 2C**). To confirm the effect of ISG12 on ERα protein levels we analyzed total protein extracts from MCF-7 and MCF7-ISG12 cells grown with or without E2 by Western blot. The results showed that E2 treatment increased by 20% the ERα protein levels in control MCF-7 cells (p < 0.05) (**Figure 3A**, control MCF-7). In contrast, MCF7-ISG12 cells treated with E2 exhibited a 35% reduction (p < 0.05) in ERα protein levels with respect to MCF7-ISG12 cells grown in hormone-free medium and a 46% reduction (p<0.01) with respect to ERα protein levels in MCF-7 cells treated with E2 (**Figure 3A**, MCF7-ISG12). To determine whether the effect of ISG12 on ERα protein levels is specific, we transfected MCF-7 cells with the vector pCMV-TTP encoding the nuclear hormone corepressor tristetraprolin (TTP) (19) to compare its effect on ERα transactivation and protein levels. The results showed that TTP transfection reduced ERα activity by 80% in MCF-7 cells stimulated with E2 (p < 0.01) (**Figure 3B**). However, densitometric quantification of Western blot bands revealed that TTP over-expression did not reduce ERα protein levels (**Figure 3C**). These results suggest that while both TTP and ISG12 expression down-regulate ERα transactivation, only ISG12 reduces ERα protein levels in breast cancer MCF-7 cells.

**Figure 4:**
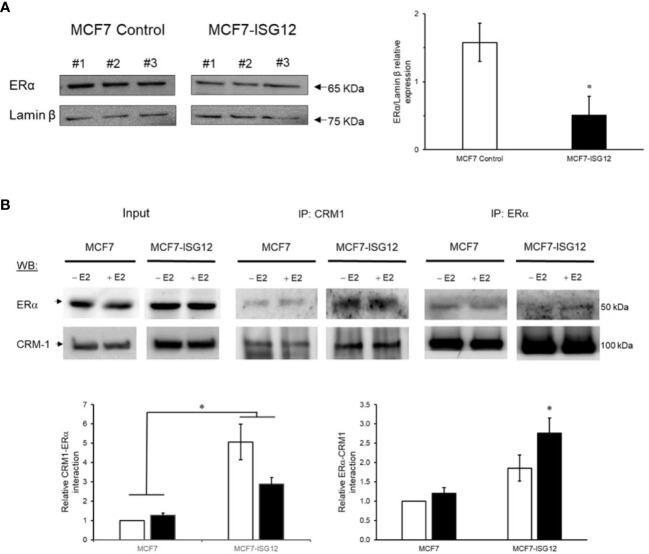
ISG12 over-expression reduces ERα nuclear protein levels. **(A)** Nuclear protein extracts prepared from E2 stimulated control MCF-7 and MCF7-ISG12 cells nuclei were resolved by PAGE, and expression of ERα and lamin β, as a loading control protein, were evaluated by Western blot. Results from densitometric analysis of protein bands from three different experiments are represented as mean S.E. Differences in ERα protein levels between control MCF-7 and MCF7-ISG12 cells were shown to be statistically significant (p < 0.05). **(B)** ISG12 increase the interaction between ERα and CRM1/XPO1. MCF-7 and MCF7-ISG12 nuclear protein extracts were immunoprecipitated with anti-ERα antibody (IP: ERα) or anti-CRM1 antibody (IP: CRM1) followed by WB with anti-CRM1 or anti-ERα. Densitometric analysis of protein bands from two different experiments are represented as mean S.E. Differences in CRM1-ERa interaction levels between control MCF-7 and MCF7-ISG12 cells were shown to be statistically significant (p < 0.05). Input lanes represents 10% of the nuclear extract used in the capture assays.*p < 0.05.

Next, we focus on the effect of ISG12 expression on ERα located in the cell nucleus where it is responsible for the estradiol-dependent transcriptional regulation. Western blot analysis was performed on protein extracts prepared from isolated nuclei from control MCF-7 and MCF7-ISG12. Our results showed that ERα nuclear protein levels in MCF7-ISG12 cells were 67% lower (p < 0.01) than in control MCF-7 cells (**Figure 4A**). To explore whether ISG12 down-regulates ERα transactivation by promoting its nuclear export, we used co-immunoprecipitation assays to determine the interaction of ERα with the nuclear exportin protein chromosomal maintenance 1 (CRM1/XPO1). Nuclear protein extracts for control MCF-7 or MCF7-ISG12 cells gown in hormone free medium or in E2 supplemented medium were immunoprecipitated using anti-CRM1 antibody. The precipitated proteins were separated in acrylamide gels and the interaction with ERα was determined by densitometric analysis of Western blots bands. Our results showed that ISG12 overexpressing MCF-7 cells incubated in hormone free or E2 supplemented medium exhibit a 500% and 300% increase (p < 0.05), respectively, in the interaction between CRM1/XPO1and ERα compared to control MCF7 cells (**Figure 4B**, IP: CRM1). The effect of ISG12 on the interaction between ERα and CRM1/XPO1 was confirmed by a reciprocal coimmunoprecipitation assay in which nuclear protein extracts from MCF-7 cells and MCF7-ISG12 cells were immunoprecipitated with anti-ERα antibody and the interaction with CRM1/XPO1was visualized by Western blot using anti-CRM1 antibody (**Figure 4B**, IP: ERα). These experiments showed an 200% and 270% increase (p < 0.05) in the interaction between ERα and CRM1/XPO1 in MCF7-ISG12 cells grown in hormone-free and E2 medium with respect to control MCF-7 cells. As a control, 10% of the protein extracts used in each immunoprecipitation assay were analyzed by Western blot using anti- CRM1/XPO1or anti-ERα to confirm the presence of the proteins (**Figure 4B**, Input). In combination, these results indicate that ISG12 promotes the interaction of ERα with the nuclear exportin CRM1/XPO1 and suggest that the ISG12-dependent down-regulation of ERα transactivation may be mediated by its export from the nucleus in breast cancer cells.

### ISG12 Impairs the Migration of Breast Cancer Cells

**Figure 5:**
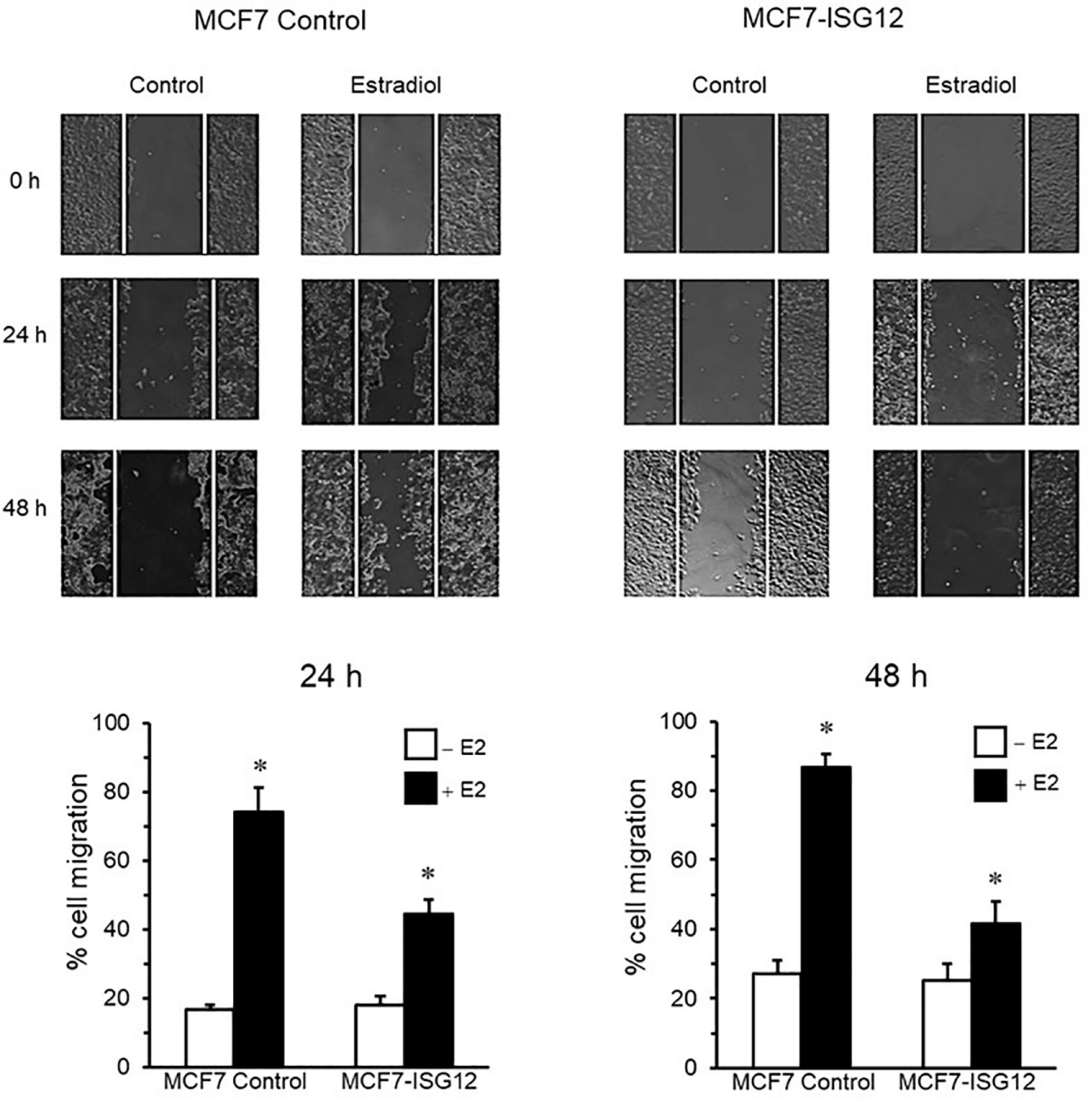
ISG12 over-expression inhibits the migration of MCF-7 cells. Control MCF-7 and MCF7-ISG12 cells were grown to confluence and incubated for 24 or 48 h in the absence (−E2) or presence (+E2) of estradiol. The migration of the cells was determined by the wound-healing assay as described in *Materials and Methods*. Results are represented as mean ± S.E of three independent experiments. Differences in MCF-7 and MCF7-ISG12 cells migration in the presence of E2 were shown to be statistically significant (p < 0.01). *p < 0.01.

To start assessing the physiological impact of ISG12 over-expression in breast cancer we compared the migration ability of control MCF-7 cells and MCF7-ISG12 cells. For these experiments we used the wound-healing assay which allows to determine the migration potential of cancerous cells. Our results showed that control MCF-7 cells stimulated with E2 exhibited a 60% increase in motility at 24 and 48 h compared to MCF-7 cells grown in hormone-free medium (p < 0.01) (**Figure 5**, MCF-7 panel). In contrast, the presence of ISG12 impaired the migration of MCF-7 cells. MCF7-ISG12 cells incubated in E2 for 24 and 48 h exhibited an increase of only 45% and 40% (p < 0.01) compared to MCF7-ISG12 cells incubated in hormone-free medium (**Figure 5**, MCF7-ISG12 panel).

### ISG12 Over-Expression Reduces Proliferation of Breast Cancer MCF-7 Cells

**Figure 6:**
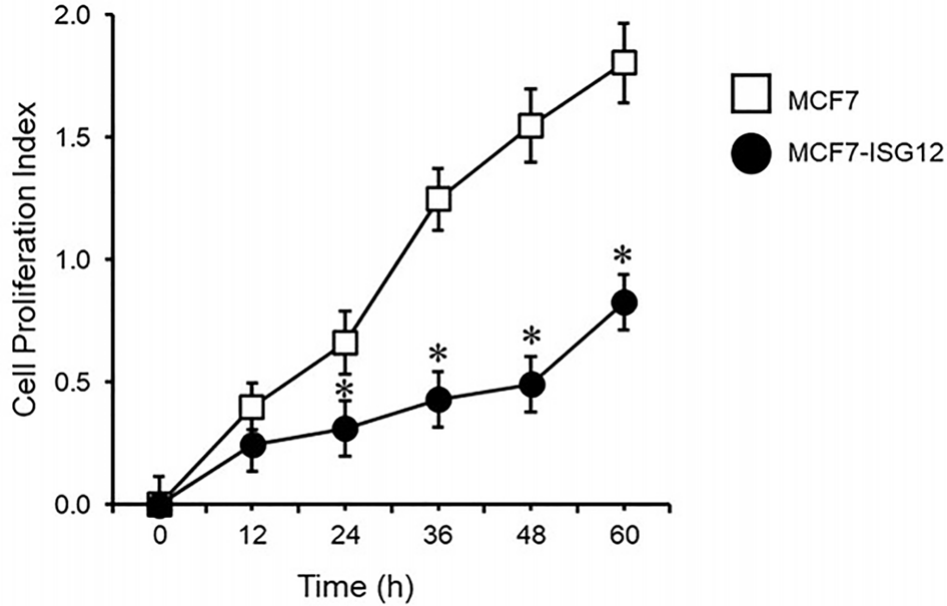
ISG12 impairs the proliferation of MCF-7 cells. Control MCF-7 and MCF7-ISG12 cells were grown in the presence of estradiol and proliferation was recorded on the xCELLigence RTCA System in real-time every 30 min, for 60 h as described in *Materials and Methods*. Results are represented as mean ± S.E of six independent experiments. *p < 0.01.

Given that ERα is the major driver of cell proliferation in breast cancer and ISG12 was identified as an over-expressed protein in different breast cancer cell lines, we explored whether MCF7-ISG12 cells proliferate at a different rate than control MCF7 cells. For this assay 7.5 × 10^3^ control MCF-7 or MCF7-ISG12 cells were cultured in the presence of E2, and cell proliferation was determined using the Xcelligence RTCA, ACEA Bioscience (Roche). Although at 12 h, the proliferation of control MCF-7 and MCF-7-ISG12 cells were similar, after 24, 36, 48 and 60 h, the number of MCF7-ISG12 cells was 49%, 34%, 31%, and 45% lower, respectively, than the number of control MCF7 cells (p < 0.05) (**Figure 6**). These results suggest that ISG12 overexpression reduces the rate of cell division in breast cancer cells.

### ISG12 Expression Reduces Spheroid Formation, Estradiol-Dependent Cell Proliferation and Ki67 Expression in 3-D Cell Cultures

To explore further the biological relevance of ISG12 expression on cell proliferation we examined the effect of increasing ISG12 protein levels on 3-D cell cultures. This system allows cells to grow in a microenvironment that mimics cell-cell and cell-matrix interactions and nutrient transport gradient dynamics that exist in living tissues. Control MCF-7 and MCF7-ISG12 cells were grown for 14 days atop a reconstituted layer of Matrigel to form spheroids and incubated in hormone-free medium or in medium containing 100 nM E2 or 1 μM TOT. The 3-D cultures were incubated with anti-Ki67 antibody. The proliferation marker Ki67 reflects the tumor cell proliferation rate as it correlates with progression, metastasis and prognosis in a number of different malignancies and is widely used in routine clinicopathological investigation (40). In this study, the number of Ki67-positive cell nuclei was used as an indicator of cell proliferation.

**Figure 7:**
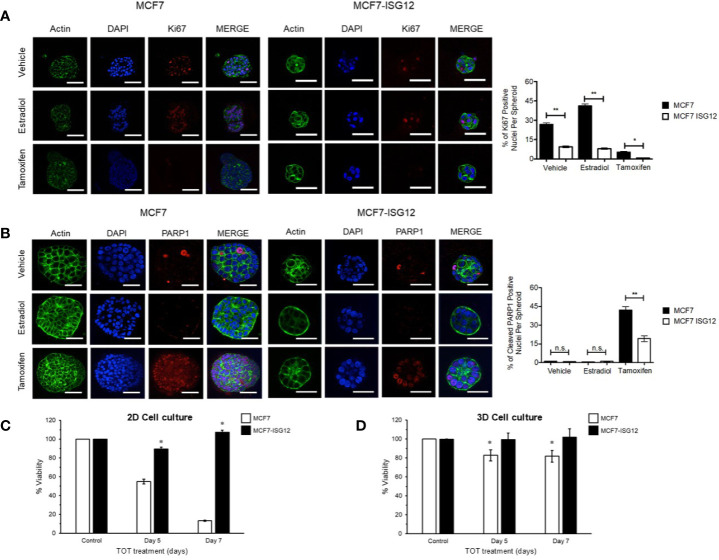
ISG12 inhibits estradiol-dependent proliferation and tamoxifen-induced apoptosis in MCF-7 3-D cultures. Control MCF7 and MCF7-ISG12 cells were grown for 14 days in matrigel to form spheroids and incubated in hormone-free medium (vehicle) or in medium containing 100 nM E2 or 1 µM TOT. The structure of spheroids was visualized by incubation with DAPI (blue) and anti-actin antibody (green). To determine the effect of ISG12 on cell proliferation **(A)** and apoptosis **(B)**, the 3-D cultures were incubated with anti-Ki67 **(A)** or anti cleaved PARP1 (PARP1) antibodies. The number of Ki67 or PARP1 positive nuclei in spheroids were taken as indicators of cell proliferation or apoptosis, respectively. Results are represented as mean ± S.E. of three independent experiments and were shown to be statistically significant (*p < 0.05; **p < 0.01). The effect of TOT on cell viability of monolayer **(C)** and 3-D cultures **(D)** of control MCF-7 (white bars) and MCF7-ISG12 cells (black bars) was determined as described in *Material and Methods*. Results are shown as mean ± S.E. and the differences between MCF-7 and MCF7-ISG12 spheroids were shown to be statistically significant (p < 0.05). Scale bar, 50 μm. n.s., not statistically significant.

The spheroids formed by control MCF-7 cells were characterized by exhibiting a compact structure and defined external borders (**Figures 7A, B**, actin and DAPI panels). Incubation with anti–Ki-67 antibody identified that 27% of the total number of cells in control MCF-7 spheroids incubated in hormone free medium were positive for Ki67 (**Figure 7A**, Ki67, vehicle). Stimulation with E2 increased to 43% (p < 0.05) the percentage of proliferating Ki67 positive cells in control MCF7 spheroids (**Figure 7A**, Ki67 estradiol panel). As expected, treatment of ERα-expressing control MCF7 spheroids with the anti-estrogen TOT reduced the percentage of Ki-67–positive nuclei in spheroids to 4% (p < 0.01) (**Figure 7A**, Ki67 tamoxifen panel). In contrast, ISG12 overexpressing MCF-7 spheroids exhibited a less compact structure and irregular borders (**Figure 7A**, actin and DAPI panels). Anti-Ki67 staining demonstrated that in MCF7-ISG12 spheroids incubated in E2 medium only 10% of the cells were positive for Ki67. Further, no significant differences in the percentage of Ki67 positive nuclei were observed in MCF7-ISG12 spheroids incubated in hormone-free or E2 medium (**Figure 7A**, vehicle and estradiol panels). In the presence of TOT only 1% of the nuclei was positive for the expression of Ki67 spheroids. These results indicate that ISG12 overexpression impairs spheroid formation and reduces the estradiol-dependent Ki67 expression and cell proliferation in breast cancer 3-D cultures.

### ISG12 Expression Reduces Tamoxifen-Induced Apoptosis in 3-D Cell Cultures

In cancer, tumor growth and progression could result from an increase in cell proliferation or inhibition of apoptosis or a combination of both. Because in mammary gland tissue ERα activation affects both proliferation and apoptosis we explored the effect of ISG12 on tamoxifen-induced apoptosis. Control MCF-7 or MCF7-ISG12 spheroids were incubated in hormone-free medium or in medium containing 100 nM E2 or 1 μM TOT for 1 h. The 3-D cultures were incubated with an antibody directed against the cleaved form of PARP1 (Asp214) which is a product of caspases activity and considered a hallmark of apoptosis (41). Cleaved PAPR1 positive cell nuclei were used as an indicator of the number of apoptotic cells. The results showed that less than 1% of cells in control MCF-7 spheroids incubated in hormone-free or E2 supplemented medium were positive for cleaved PARP1 (**Figure 7B**, MCF-7 vehicle and estradiol panels). As expected, in the presence of TOT the number of apoptotic cells in MCF-7 spheroids increased to 45% (p < 0.01) (**Figure 7B**, MCF-7 tamoxifen panel). In MCF7-ISG12 spheroids treated with vehicle or E2 less than 1% of the cells were positive for cleaved PARP1. In contrast, treatment with TOT increased the number of apoptotic cells in MCF7-ISG12 spheroids to 18% (p < 0.01). These results suggest that ISG12 expression reduced by almost 50% the efficiency of TOT to induce apoptosis compared to control MCF-7 3-D cell cultures.

To confirm the ISG12 inhibition of TOT-induced apoptosis we determined the viability of 2-D and 3-D cultures of control MCF-7 and MCF7-ISG12 cells treated with 5 µM TOT for 5 or 7 days. In 2-D cultures, TOT treatment for 5 and 7 days reduced the viability of control MCF-7 cells by 65% and 90% (p < 0.05), respectively compared to control cells (**Figure 7C**, white bars). Instead, the viability of 2-D cultures of MCF7-ISG12 cells incubated in the presence of TOT showed a reduction of only 10% at 5 days and an increase in the cells viability of 8% at 7 days of treatment (p < 0.05) compared to control MCF-7 cells (**Figure 7C**, black bars). When we exposed 3-D cell cultures to TOT the viability of control MCF-7 spheroids was reduced by 20% and 25% after 5 and 7 days of treatment (p < 0.05) (**Figure 7**, black bars D). In contrast, the viability of ISG12 over-expressing spheroids was not affected by TOT (**Figure 7**, black bars D). These results in combination with Ki-67 and cleaved PARP1 immunostaining experiments suggest that ISG12 overexpression impairs the estradiol signaling pathway that is responsible for both the E2-dependent proliferation and the TOT-induced apoptosis in breast cancer cells.

### Correlation Between ISG12 mRNA Expression Levels and Relapse-Free Survival in Breast Cancer Patients

**Figure 8:**
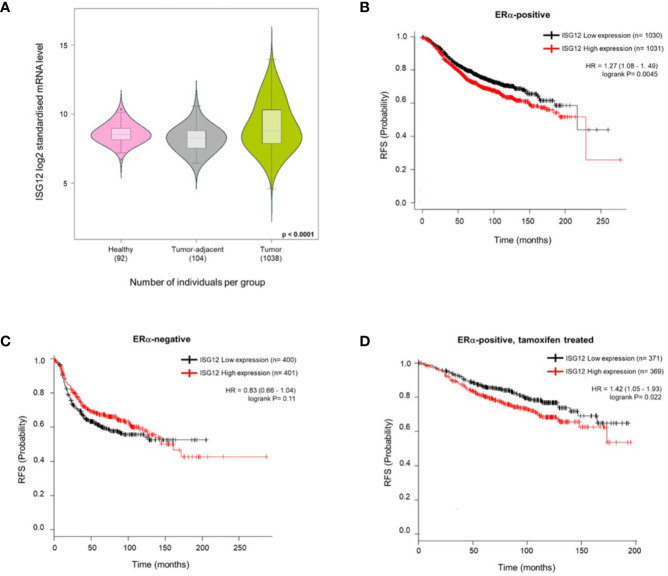
ISG12 expression in breast cancer tumors and Kaplan-Meier analysis of the association between ISG12 expression and relapse-free survival (RFS) in breast cancer patients. **(A)** ISG12 mRNA expression levels in normal and breast cancer tissue was determined using the Breast Cancer Gene-Expression Miner database. The results are shown as a violin plot of the log2 of ISG12 mRNA expression (p = 0.0001, Dunnett-Tukey-Kramer’s test). **(B)** Patients with ERα-positive breast cancer tumors and high ISG12 expression levels had poor RFS. **(C)** patients with ERα-negative breast cancer tumors had no significant association between ISG12 expression and RFS. **(D)** Patients with ERα-positive breast cancer tumors and high ISG12 expression levels that received tamoxifen as part of their treatment had poor RFS.

To explore the relationship between ISG12 expression with breast cancer tumorigenesis, we made use of the Breast Cancer Gene-Expression Miner database (http://bcgenex.centregauducheau.fr/BC-GEM/GEM-Accueil.php?js=1) to compare the ISG12 mRNA levels in tumors, adjacent-tumor tissue and normal tissue in breast cancer patients. The results showed that ISG12 mRNA levels are increased up to 140-fold in tumors compared to tumor-adjacent tissue or healthy tissue (p = 0.0001, Dunnett-Tukey-Kramer’s test) (**Figure 8A**). Next, we used the KM-ploter database to compare the expression levels of ISG12 mRNA in breast cancer tumors. The results were ranked from low to high based on ISG12 mRNA median values. Kaplan-Meier curves were generated to compare relapse-free survival (RFS) between the low and high expression groups. The results showed that patients with ERα positive and high ISG12 mRNA expression tumors had significantly worse relapse*-*free survival than patients with low ISG12 expression levels (**Figure 8B**). In patients with ERα negative tumors the RFS rate did not show a significant association to ISG12 expression levels (**Figure 8C**) suggesting that the effect of ISG12 on RFS in breast cancer patients depends on the expression of ERα. Interestingly, when we focused on ERα positive breast cancer patients that had received tamoxifen as part of their treatment we found that individuals with high ISG12 mRNA expression levels had a significantly worse relapse*-*free survival rate than patients with low ISG12 expressing tumors (**Figure 8D**).

## Discussion

In this work we identified ISG12 as a novel ERα-associated protein using a two-step selection protocol consisting in a yeast two-hybrid screen followed by transient transfection assays in human breast cancer cells. We had previously used this experimental strategy to identify nuclear receptor coregulators including NHERF2 and TTP (19, 20). ISG12 is 122 amino acid protein that belongs to a family of hydrophobic proteins designated FAM14 (42), characterized by possessing a highly conserved 80 amino acid domain known as the ISG12 motif (43). ISG12 was originally identified as a cell factor localized in the nuclear envelope and whose expression is induced by estradiol and interferon in different human breast cancer cell lines (37). More recent studies have reported that ISG12 is over-expressed at the mRNA or protein levels in primary invasive breast carcinomas, breast cancer bone metastasis, oral squamous cell carcinoma, psoriatic epidermis, chronic eczema and cutaneous squamous cell cancers (37, 38, 44). The function and impact of ISG12 expression and over-expression in human cells is not completely understood, but functional and molecular studies suggest it may have different roles in cell physiology.

Using Proximity ligation assays (PLA), a technique that allows the detection of proteins and proteins interactions with single molecule resolution, we confirmed that endogenously expressed ISG12 is an ERa associated protein in human MCF-7, T47-D, and ZR-75-1 cells. The overexpression of ISG12 was shown to attenuate ERα transactivation through different experimental approaches. First, exogenously expressed ISG12 reduced both the estradiol dependent and independent ERα transcriptional activity in a dose-dependent manner in transient transfection assays in human breast cancer MCF-7 and T47D cells. Second, ISG12 overexpressing MCF-7 cells showed a reduction in the mRNA levels of ERα target genes cathepsin D and GREB1 compared to control MCF-7 cells.

The mechanism responsible for the ISG12 effect on ERα transcriptional activity is different from that exhibited by nuclear receptor coregulators. For example, coactivators, like SRC-1, GRIP1/TIF2, and NHERF2 are localized to the nuclear compartment where they interact with the AF2 region of nuclear receptors as part of large coactivator protein complexes that possess histone acetyl transferase activity (20, 45). In the same way, the corepressors NCOR, SMRT, and TTP also require to be translocated inside the cell nucleus to interact with nuclear receptors and repress their transactivation activity as part of corepressor protein complexes that exhibit histone deacetylase enzymatic activity (19, 46–48).

The regulatory function of ISG12 seems to be atypical because, unlike *bona fide* corepressors, it is not highly expressed in the cell nucleus and PLA and coimmunoprecipitation experiments showed it co-localizes more abundantly with ERα in the cytoplasm and perinuclear region than in the cell nucleus. Further, immunostaining of MCF-7 cells and Western blot analysis of total cell protein extracts and nuclear protein extracts showed that ISG12 overexpression is accompanied by a reduction in nuclear ERα protein levels in MCF7 cells. To test the specificity of these results we transfected MCF-7 cells with the nuclear receptor coregulator TTP. We had previously shown that TTP represses the transactivation activity or different steroid nuclear receptors including ERα, progesterone receptor, androgen receptor and glucocorticoid receptor without affecting their protein levels in breast cancer cells (49). Our results showed that TTP transient transfection into MCF-7 cells reduced ERα transactivation and Western blot analysis of protein extracts confirmed that TTP expression in MCF-7 cells did not reduce ERα protein levels suggesting that the ISG12 effect on its nuclear protein levels is specific.

Our results suggest that ISG12 reduces ERα nuclear protein levels by facilitating its interaction with CRM1/XPO1. In human cells the nuclear localization and expression levels of RNA molecules, transcription factors, oncoproteins and tumor suppressor proteins is the result of a delicate balance between import to the nucleus and export to the cytoplasm through the nuclear pore complex of a cell. CRM1/XPO1 is the major receptor for the export of proteins, including ERα and other hormone nuclear receptors, out of the nucleus (31, 35, 50). In human cells the CRM1/XPO1-dependent export of ERα has been shown to regulate its nuclear protein levels and to reset the steroid signal transcription pathway by preventing the nuclear accumulation of transcriptionally inactive forms of ERα that after their export from the nucleus are degraded by the proteasome (35). The functional impact of ISG12 on ERα was demonstrated by our experiments that show that ISG12 overexpression reduces both the E2-dependent and E2-Independent ERα transactivation activity and the expression of genes transcriptionally regulated by estradiol. These changes likely reflect the reduction in the nuclear protein levels of transcriptionally active ERα in MCF-7 and T47-D cells.

Our results on the effect of ISG12 on the physical interaction between ERα and CRM1/XPO1 partially replicate those previously reported by Papac-Milicevic et al. (51) This group showed that ISG12 localized in the nuclear envelop interacts with the orphan nuclear receptor NR4A1 and promotes its nuclear export in a CRM1/XPO1 dependent manner reducing the expression of its target genes. These findings and the ISG12-dependent downregulation of ERα transactivation suggest that ISG12 forms part of a general regulatory mechanism that modulates the transactivation activity of multiple hormone nuclear receptors by facilitating their exit from the cell nucleus *via* the CRM/XPO1 complex.

It has been suggested that the dysregulation of the cellular balance of nuclear receptor associated proteins is linked to the development of different forms of cancer. For example, increased expression the coactivators SRC-1 and NHERF2 or the loss of expression of corepressors such as NCoR and TTP correlate with cell proliferation, tumor development and progression (19, 20, 34, 52). Similarly, changes in the expression levels of proteins that affect ERα nucleocytoplasmic translocation such as CRM1/XPO1 or prosaposin have also been associated to breast cancer tumor development (3). In particular CRM1/XPO1 overexpression in breast cancer tumors is associated to poor prognostic characteristics including larger tumor size and positive lymph node metastasis (53).

In recent years different research groups have documented the association between increased ISG12 expression and different forms of cancer. The impact of ISG12 in tumor development is not clearly understood because the observed effects of ISG12 over-expression are quite diverse and, in some cases, antagonistic in nature. For example, in ovarian cancer, psoriatic skin, cutaneous squamous cell cancers and cholangiocarcinoma patients ISG12 over-expression has been associated to epithelial proliferation, epithelial–mesenchymal transition, cell cycling and tumorigenicity (38, 54–56). In contrast, other studies have linked ISG12 to control of the innate immune response and regulation of IFN-induced apoptosis (42, 57, 58). These studies have determined ISG12 overexpression using different cancer cell lines in culture or by analyzing cDNA microarray databases, and it is not known whether these different outcomes are result from tissue-specific mechanisms or by differences in ISG12 protein expression levels.

In this work we have studied the effect of ISG12 under conditions of protein overexpression in breast cancer cells and the results suggest that increased ISG12 levels lead to an augmented export of ERα from the nucleus. It is possible that the reduction in the nuclear ERα protein levels impairs the steroid signal transduction pathway which in breast cancer cells is responsible for the E2-dependent cell proliferation and TOT-induced apoptosis (59). This hypothesis seemed to be confirmed by the observation that in the presence of increased levels of ISG12 E2 and TOT were less effective to induce cell proliferation or to reduce the number of viable cells, respectively in 2-D and 3-D cell cultures.

One of the main causes of mortality in breast cancer patients is the development of estradiol-independent and tamoxifen-resistant tumor growth. Although this is believed to be a multifactorial phenomenon our results suggest the possibility that the ISG12 mRNA overexpression reported in human breast carcinomas may contribute to the impaired hormonal response in breast cancer cells.

To explore a possible relation between ISG12 overexpression levels and development of breast cancer we analyzed public mRNA microarray databases. These experiments showed that breast cancer patients that received treatment with TOT and whose tumors exhibited high ISG12 mRNA levels had a significant reduction in RFS with respect to patients with low expression levels of ISG12 that also received this anti-estrogen therapy. Our results cannot exclude the possibility that the apparent effect of ISG12 on RFS in breast cancer patients could be associated to the dysregulation of the nuclear protein levels of yet to be identified tumor suppressors and other transcription factors whose nuclear export is mediated by the CRM/XPO1 system. However, our findings on the effect of ISG12 on the response of breast cancer cells to E2 and TOT suggest the need to explore further whether ISG12 protein overexpression could impair the cellular response to E2 and TOT which is often observed in tumor recurrence and metastatic breast cancer (60–62).

In summary, this study has identified ISG12 as a novel ERα-associated protein that participates in the nuclear to cytoplasm export of this hormone nuclear receptor by facilitating its interaction with the exportin CRM1/XPO1. In normal cells ERα nuclear export plays an important role in the control of its nuclear protein levels and in preventing the nuclear accumulation of inactive ERα proteins which in the cytoplasm are eventually degraded by the proteasome. The characterization of ISG12 as a facilitator of the interaction between CRM1/XPO1 and ERα will help to a better understanding of the impact of nuclear to cytoplasm transport on the regulation of ERα transactivation. The impairment in the cellular E2-dependent proliferation and TOT-induced apoptosis by ISG12 over-expression suggest the possibility that this protein affects proliferation, migration and response to hormonal treatment in breast cancer cells. Further studies will be necessary to explore the relationship between increased ISG12 protein levels in breast cancer patients with tumor progression, metastatic disease and to explore the potential of ISG12 expression levels in the development of new diagnostic and therapeutic strategies.

## Data Availability Statement

The raw data supporting the conclusions of this article will be made available by the authors, without undue reservation.

## Author Contributions

MC-B performed qPCR, WB, and Co-IP experiments and participated in the writing of the article. AP-V performed cell transient transfection, luciferase reporter assays, bioinformatic and statistical analysis and participated in the writing of the article. VG-R and RC-R performed MCF-7 and MCF7-ISG12 Immuno-staining, cell proliferation and wound-healing assays and stable transfection assays. AC-Q contributed in cell proliferation assays. LA-R and OV-C designed and performed immunostaining and confocal microscopy experiments of MCF-7 and MCF7-ISG12 3-D cultures and ISG12 PLA assays. MG-M collaborated in nuclear CRM1 co-IP experiments. GR-G and TB-G performed TTP cell transfection assays and TTP effect on ERα protein levels. LC-V performed TOT effect on 3-D cultures. AZ-D contributed to conception and design of study and analysis of results. AL-D-R contributed to conception, design of study and analysis of results, performed yeast two-hybrid cDNA library screen, and wrote the final draft of the article. All authors contributed to the article and approved the submitted version.

## Funding

This work was funded by grants from Consejo Nacional de Ciencia y Tecnología (Ciencia Básica CB236405; CONACyT-Problemas Nacionales 2017-01-4900; Programa de apoyo a Proyectos de Investigación e Innovación Tecnológica, UNAM PAPIIT IV200218 and PAPIIT IN208018.

## Conflict of Interest

The authors declare that the research was conducted in the absence of any commercial or financial relationships that could be construed as a potential conflict of interest.
